# Participant Motivators and Expectations in the MEL-SELF Randomized Clinical Trial of Patient-Led Surveillance for Recurrent Melanoma: Content Analysis of Survey Responses

**DOI:** 10.2196/58136

**Published:** 2024-10-17

**Authors:** Deonna Ackermann, Jolyn Hersch, Dana Jordan, Emily Clinton-Gray, Karen Bracken, Monika Janda, Robin Turner, Katy Bell

**Affiliations:** 1 School of Public Health Faculty of Medicine and Health The University of Sydney Sydney Australia; 2 Kolling Institute Faculty of Medicine and Health The University of Sydney Sydney Australia; 3 Centre for Health Services Research The University of Queensland Brisbane Australia; 4 Biostatistics Centre University of Otago Dunedin New Zealand

**Keywords:** teledermatology, melanoma, randomized controlled trial, trial recruitment and retention, studies within a trial, SWATs, dermatology, cancer, early detection, dermatology clinical trials, clinical trials, mobile phone

## Abstract

**Background:**

Limited data exist on the motivations and expectations of participants when enrolling in dermatology clinical trials, including melanoma early detection trials. Understanding participant motivators for research engagement has been identified as a prioritized area for trial methodology research.

**Objective:**

The study aimed to determine motivators of participation and expectations from trial involvement among patients enrolled in the MEL-SELF randomized clinical trial of patient-led surveillance for new or recurrent melanoma.

**Methods:**

The MEL-SELF trial is recruiting patients previously treated for localized melanoma, who own a smartphone, have a partner to assist with skin self-examination (SSE), and attend routinely scheduled follow-up at specialist and primary care skin clinics in Australia. We evaluated responses from the first 100 randomized participants to 2 open-ended questions about their motivations and expectations for participating in the trial, administered through the internet-based baseline questionnaire. A total of 3 coders independently coded the free-text responses and resolved discrepancies through consensus. Qualitative content analysis by an iterative process was used to group responses into themes. Responses from potential participants who were not randomized and the 404 participants randomized subsequently into the trial, were also checked for new themes. Coding and analysis were conducted in Microsoft Excel.

**Results:**

Out of the 100 survey participants, 98 (98%) answered at least 1 of the 2 questions. Overall, responses across the motivation and expectation items indicated 3 broad themes: community benefit, perceived personal benefit, and trusting relationship with their health care provider. The most common motivators for participation were related to community benefit. These included progressing medical research, benefitting future melanoma patients who may have similar experiences, and broader altruistic sentiments such as “helping others” or “giving back.” The most common expectations from the trial related to personal benefit. These included perceived improved outcomes such as earlier diagnosis and treatment, access to additional care, and increased self-empowerment to take actions themselves that benefit their health. Patients expressed a desire to gain health-related knowledge and skills and were interested in the potential advantages of teledermatology. There were no new themes in responses from those who were not randomized or were randomized subsequent to the first 100.

**Conclusions:**

We report a tailorable, patient-focused approach to identify drivers of research engagement in clinical research. Clinical trials offer an opportunity to collate a substantial evidence base on determinants of research participation and to identify context-specific factors. Results from the MEL-SELF trial emphasized notable altruism, self-empowerment, and perceived advantages of teledermatology as specific motivators. These findings informed consent processes, recruitment, retention, response to trial tasks, and intervention adherence for the MEL-SELF host trial.

**Trial Registration:**

Australian New Zealand Clinical Trials Registry (ANZCTR): ACTRN12621000176864. https://www.anzctr.org.au/Trial/Registration/TrialReview.aspx?id=379527&

## Introduction

Participants’ willingness to engage with research is an important determinant of study feasibility. Research engagement in randomized clinical trials (RCTs) involves successful recruitment, consenting, retention, response to trial tasks, and adherence to intervention and control conditions. Challenges are often reported across these participation components, resulting in research waste (suboptimal recruitment, response to trial tasks, and adherence can result in underpowered and inconclusive studies), increased costs, and delayed availability of potentially effective interventions for patients [1]. However, the evidence base for effective strategies to improve research engagement, including in dermatology research, is sparse [2-4].

Identification and understanding of the motivators and expectations of participants in clinical trials may provide trialists with the knowledge required to develop strategies that facilitate research engagement [5]. The PRioRiTy (Prioritizing recruitment and retention in randomized trials) study, a James Lind Alliance Priority Setting Partnership, concluded that one of the most pressing recruitment questions is to determine what motivates trial participation [6] and that the top retention priority is to understand what motivates participants to complete a clinical trial [7].

A recent overview of systematic reviews highlighted perceived personal benefits as the most commonly reported motivator for research participation, with other key motivators including altruism, trust in the clinician, low burden, and financial incentives [8]. The included reviews were from specific clinical specialties, including advanced cancer management, HIV, mental health, chronic obstructive pulmonary disease, and emergency medicine. Some focused on specific populations such as children, pregnant women, older adults, and ethnic minority groups. However, a gap exists in the determinants of research participation in dermatology trials and melanoma surveillance in particular.

The MEL-SELF RCT aims to assess whether patient-led surveillance (comprising smartphone-supported skin self-examination, teledermatology, fast-tracked unscheduled clinic visits in addition to routinely scheduled clinic visits) compared with clinician-led surveillance (usual care) leads to increased diagnoses of a new primary or recurrent melanoma ahead of routinely scheduled clinic visits [9]. The intervention was tested in a pilot RCT, which identified difficulties with participant engagement across a variety of trial processes [10].

To inform the design of strategies to improve participant engagement in the larger ongoing MEL-SELF trial, we asked participants to provide free text responses about why they wanted to participate in the trial and what they hoped to get out of it. The questions were administered as part of the internet-based baseline questionnaire before randomization into the trial. This Study Within A Trial (SWAT, an embedded research study within a clinical trial aimed at evaluating and optimizing trial design, processes, or interventions) [2] aimed to identify relevant information to optimize ongoing recruitment, retention, trial task response, and adherence processes both within the current MEL-SELF trial and future dermatology clinical trials. Furthermore, we aimed to determine whether research engagement motivators are consistent with previously identified themes in other disease areas [8] and whether there are important context-specific factors.

## Methods

### Participants and Setting

The MEL-SELF RCT recruits patients attending routine melanoma follow-up from specialist-led and primary care skin cancer clinics in Australia [10]. Eligible participants have been previously treated for localized melanoma (American Joint Committee on Cancer stage 0, I or II), own a smartphone, have a partner to assist with skin self-examination (SSE), are able to understand English, and have no documented history of cognitive impairment. Patients are provided with information about the trial by their doctor, and permission is given to researchers to contact them. Researchers email additional information, including a patient information sheet and a link to sign an electronic informed consent form. Potential participants enter an active run-in phase, which requires them to complete the internet-based baseline questionnaire, view instructional videos, complete electronic reporting of their SSE findings, and upload macroscopic digital photos of 1 melanocytic skin lesion to the study’s web-based platform. Participants who complete these tasks are randomized into the trial. Approval to conduct the study was granted by the Sydney Local Health District (RPAH zone) Ethics Review Committee.

This embedded SWAT includes the first 100 participants randomized into the MEL-SELF trial who were recruited from 3 skin cancer clinics in Sydney and Newcastle, New South Wales, Australia (2 specialist-led clinics and 1 primary care skin clinic). The protocol is available on the SWAT repository [11].

### Data Collection and Analysis

A total of 2 open-ended questions about motivations and expectations were included in the internet-based baseline questionnaire delivered through the REDCap (Research Electronic Data Capture; Vanderbilt University) survey software: “Please tell us why you decided to participate in this study?” and “Please tell us what you are hoping to get out of this study?” To avoid prompting participants to respond in a certain way, we only included these 2 open-ended questions without an accompanying participation motivation scale (as has been used in other clinical areas) [12,13]. Content analysis, which combines qualitative and quantitative methods to analyze text data, was used to identify common themes in the responses [14]. A stepwise process that combined conventional and directed content analysis was used to identify both common and context-specific elements [15].

1. Initial conventional approach: after familiarization through reading and rereading the responses from both questions, an initial sample (n=50, first 50 patients completing the baseline questionnaire during the active run-in phase) was coded by DA to identify preliminary themes and subthemes.
2. Directed approach: in parallel to this, potential coding categories were derived from a review of existing literature [8].
3. Comparison and synthesis: the themes that emerged from the data itself and those derived from existing literature were examined by members of the research team (DA, Bell K) to create an initial coding framework that incorporated both common and context-specific elements.
4. Integration and application: this framework was applied to SWAT participant responses (first 100 randomized participants), and a final framework was iteratively developed and used to examine the data for themes and subthemes. Themes were concluded through discussions among 3 authors (DA, EC, and DJ, with conflicts resolved by a fourth author, Bell K).

DA coded all data, while DJ and EC checked half each. No new subthemes emerged so the sample size of 100 was retained. Coding and analysis were conducted in Excel, discrepancies were resolved through discussion, and theme frequencies were calculated. In addition, responses from participants (n=49) who did not successfully complete the active run-in phase and were not enrolled in the trial were reviewed by DA using the developed framework. These 49 participants completed the baseline questionnaire during the same period as the 100 participants who did complete the active run-in and were enrolled. This was done to identify potential differences between those who did and did not complete the active run-in, as completing this required significant motivation and effort from the patients. Finally, we also reviewed responses from the 404 trial participants who were randomized subsequent to the first 100 to check for any new themes in the full trial sample.

### Ethical Considerations

This study was approved by the Sydney Local Health District (RPAH zone) Ethics Review Committee (2019/ETH13612).

## Results

The first 100 participants enrolled in the MEL-SELF RCT completed the baseline survey from November 4, 2021, to March 28, 2022, with 98 providing a response on participation and 97 on expectations. Patient characteristics are presented in Table 1. Respondents had a mean age of 56 years (SD 13.01, range 28-83 years), were more likely to be female (59, 59%), reside in a major city (88, 88%), and have post high school or higher education (87, 87%). Half of the participants resided in areas that were in the most advantaged socioeconomic status quintile.

**Table 1:** Baseline characteristics.

Characteristic			Total (N=100)
**Age** **(years)**			
	Mean (SD)	56.4 (13.01)	
	Minimum, maximum	28, 83	
**Sex, n (%)**			
	Male	41 (41)	
	Female	59 (59)	
**Study site, n (%)**			
	Primary care skin clinic	13 (13)	
	Specialist-led clinic	87 (87)	
**Highest melanoma substage, n (%)**			
	0	34 (34)	
	IA	52 (52)	
	IB	12 (12)	
	II combined	2 (2)	
**Country of birth, n (%)**			
	Australia	78 (78)	
	Other: English speaking	15 (15)	
	Other: non–English speaking	7 (7)	
**Indigenous status, n (%)**			
	Neither Aboriginal nor Torres Strait Islander	96 (96)	
	Aboriginal or Torres Strait Islander	1 (1)	
	Unknown	3 (3)	
**Marital status, n (%)**			
	Never married	4 (4)	
	Married	74 (74)	
	De facto or in a committed relationship	15 (15)	
	Separated or divorced	6 (6)	
	Widowed	1 (1)	
**Any children, n (%)**			
	Yes	82 (82)	
	No	18 (18)	
**Level of education, n (%)**			
	High school or leaving certificate	13 (13)	
	TAFE^a^ advanced diploma or certificate	33 (33)	
	Bachelor degree	27 (27)	
	Postgraduate degree or higher	27 (27)	
**Remoteness (based on area of residence), n (%)**			
	Major city	88 (88)	
	Inner regional	11 (11)	
	Outer regional	1 (1)	
**Socioeconomic status (based on area of residence), n (%)**			
	1 (most disadvantaged)	4 (4)	
	2	9 (9)	
	3	18 (18)	
	4	19 (19)	
	5 (least disadvantaged)	50 (50)	

^a^TAFE is a government-run system in Australia that provides education after high school in vocational areas like beauty, childcare, accounting, business, and computing.

**Figure 1:**
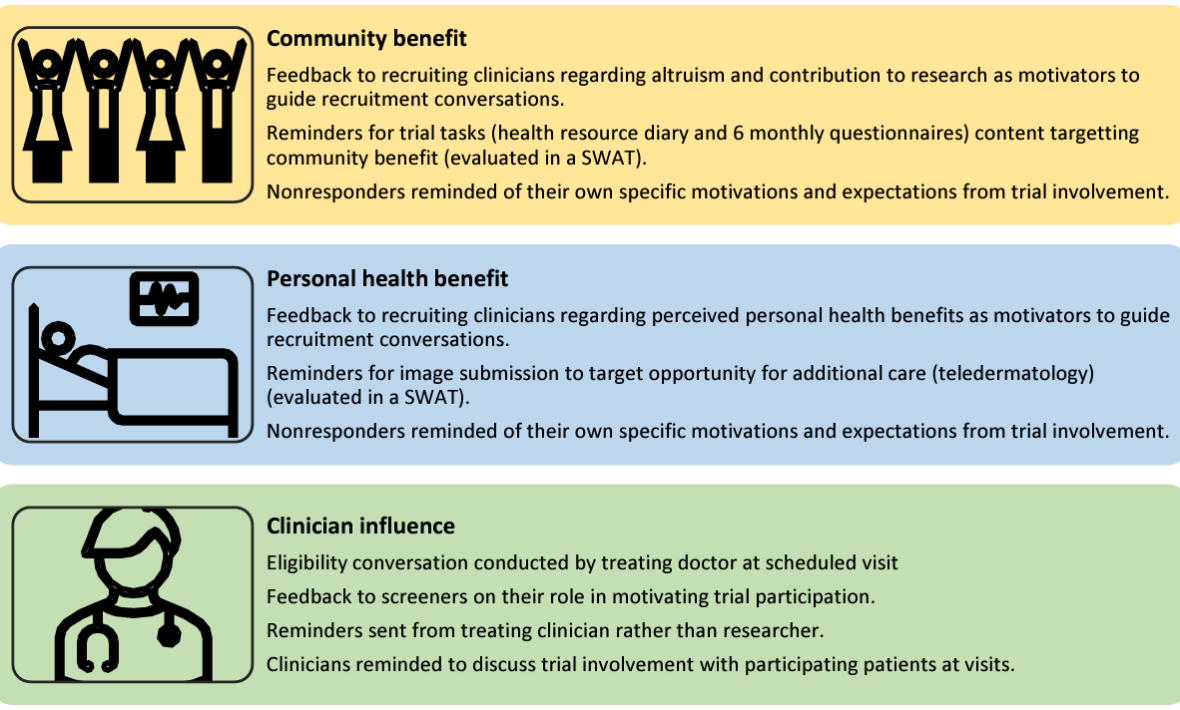
Strategies to improve trial processes implemented in the MEL-SELF trial by identified motivators of research engagement. SWAT: Study Within A Trial.

Participant responses to both questions mapped to 3 dominant themes as outlined in Table 2 and Figure 1: community benefit, personal benefit, and trust in their health care provider. Subthemes were more likely to be context-specific. Table 3 displays the frequencies of these themes and subthemes, organized by question. Participants most commonly identified community benefit as the reason for their participation, while perceived personal benefit was most commonly expressed for what they hoped to get out of the trial. A notable majority (67/100, 67%) identified community benefit as their primary motivation, followed by personal benefit (38/100, 38%) and trust in their health care provider (21/100, 21%). Regarding expectations, a significant number of participants (78/100, 78%) expressed interest in personal health benefits or insights from the trial, while community benefit was expected by 33% (33/100) participants. An additional 49 participants completed the baseline questionnaire between November 2021 and March 2022 but did not complete the active run-in phase. Out of these 100 participants, 47 (95%) provided responses to the participation and expectation questions with similar themes identified. For the participation question, 26 (53%) suggested community benefit, 17 (35%) personal benefit, and 11 (22%) trust in their health care provider. After recruitment closed (June 3, 2024), the responses from all 504 trial participants were reviewed, and no new themes emerged.

We highlight some illustrative quotes from the SWAT participants (first 100 randomized) in the sections below and present additional comments in Table S1 in Multimedia Appendix 1.

**Table 2:** Identified themes and subthemes for research engagement in the MEL-SELF randomized clinical trial (RCT).

Category and themes			Description
**Community benefit**			
	Contribution to medical research		Participants broadly recognize the importance of medical research Participants link research to improved health outcomes “Sense of duty” May express personal satisfaction arising from their contribution Participants specify a wish to contribute to advances in melanoma-related research Feel that their personal experience may particularly benefit others
	Specific merits of the intervention		Telehealth and digital technology research Benefits people who live in remote areas General
	Altruism		The trial results may benefit future melanoma patients (ie, people with a similar experience to themselves) May specify future generations of their own family Broader altruistic comments Sometimes expressed together with “nothing for me” in expectation question
**Personal health benefits**			
	Melanoma history		Beneficial due to high-risk status
	Empowerment		Increased melanoma knowledge and opportunity for learning SSE^a^ Improved skills Increased awareness of skin Increased self-confidence in SSE skills Increased self-management role: More active role in own health care Motivation Discipline Routine
	Additional care: telehealth		Access to a new intervention before it is widely available Additional access to medical services (teledermatologist review) Specific advantages of intervention for themselves Rural patients
	Improved outcomes		Earlier diagnosis and treatment Survival and quality of life
	Reassurance		Reassurance due to perceived personal health benefits
**Doctor or health facility**			
	Relationship	Clinician influence Trust in the clinician Reciprocity	

^a^SSE: skin self-examination

**Table 3:** Frequency of identified themes and subthemes for research engagement identified in 100 MEL-SELF randomized clinical trial participants^a^.

Themes	Motivators^b^	Expectations^c^
Community benefit	67	33
Contribution to medical research	30	16
Specific merits of the intervention	6	0
Altruism	36	17
Personal benefit	38	78
Perceived melanoma risk	5	0
Empowerment	18	54
Additional care	9	7
Improved outcomes	11	14
Reassurance	0	7
Trust in health care provider or facility	21	0

^a^Participants may have expressed more than 1 motivator or expectation.

^b^A total of 2 participants did not respond to the motivator question, “Please tell us why you decided to participate in this study?”

^c^A total of 3 participants did not respond to the expectations question, “Please tell us what you are hoping to get out of this study?”

### Theme 1: Community Benefit

We identified community benefit as a reason to participate in the MEL-SELF RCT in 67 responses to the first question and as a potential beneficial outcome of trial involvement in 33 responses to the second question. Subthemes included a desire to contribute to the advancement of medical research, a belief in the specific merits of patient-led surveillance, and altruism.

#### Contribution to Scientific Research

Some participants broadly recognized the importance of research and expressed a desire to advance medical knowledge. As one participant noted:

Scientific research and evidence is important for the whole community. It is important to try new techniques to improve treatments.

P56, female, age 64 years

Support for medical research was frequently voiced, with several participants commenting that they “believe in” research. Participants often expressed an understanding that the research process is necessary for advancing medical knowledge, and they expected clinical trial results to be translated into improved medical practice.

I believe it is important to be a part of research as this will lead to better health outcomes for future patients.

P32, female, age 62 years

For some, participation was linked to a “sense of duty”:

I feel that if I qualify for a medical study, it is my duty to participate. [P78, female, age 73] and as an ex-health services researcher I am interested and feel some responsibility to support research

P85, male, age 73 years

Others characterized a sense of fulfillment from trial involvement, describing it as “pleasing,” “satisfying,” and “something to be grateful for.” For many participants, the desire to contribute was directly expressed as a commitment to advancing melanoma-related research:

“*Anything to help researchers [find] out more about melanoma and treatments.”* [P95, male, age 57 years]

Some participants felt uniquely qualified to add value to the research project due to their personal experience.

With my unusual number of melanomas, my experience may provide a clue or two that may lead to greater understanding of skin cancers.

P7, male, age 66 years

Others linked their desire to further melanoma research to their personal experience, as described by a participant who wanted “to actively support research into a health issue which has directly impacted me and could again in the future” [P18, female, age 49 years].

#### Specific Merits of the Intervention

A total of 6 participants identified that the MEL-SELF intervention had specific merits that could benefit the community, including furthering telehealth and digital technology research.

If I can be a part of something that is going [to] drive technology and innovation in this space forward, then I want to be a part of it.

P38, male, age 38 years

Regarding providing options for people living in remote areas,

Seems like a great way to improve the identification of melanoma, especially for those who live remotely.

P65, male, age 50 years

#### Altruism

The ability to help others at a community level is featured in many participants’ responses. Broad altruistic reasons for participation, such as “*in the hope I will help others*” [P75, female, age 57 years], were frequently shared and were sometimes expressed together with “nothing for me” in the expectation question. *“Nothing personally other than looking to assist.”* [P40, male, age 67 years]. Participants commonly expressed a desire to benefit future melanoma patients (ie, people with a similar experience as themselves). As 1 participant noted,

If it helps others who get/have melanoma and their timely diagnosis and treatment is afforded, then I have done something.

P6, male, age 63 years

The desire to help was sometimes linked to future generations of their own family.

Taking part in the study and knowing the results may help others in the future is something I am happy to do. I have a red-headed granddaughter, and who knows, she may benefit in the future.

P63, female, age 79 years

### Theme 2: Personal Health Benefit

Personal benefit was identified as a reason to participate in the MEL-SELF RCT in 38 participant responses to the first question and as a potentially beneficial outcome of trial involvement in 78 responses to the second question. Subthemes included a perception of benefit related to their high-risk status, empowerment, access to additional care in the form of a novel telehealth intervention, improved health outcomes, and reassurance.

#### Perceived Risk of Melanoma

Some participants acknowledged that their high-risk status due to their personal or family history of melanoma motivated them to contribute to the research. As a participant described:

I believe I have a real risk of developing melanoma - both my father and older brother had multiple melanomas removed; we spent a lot of time in the sun, unprotected, as children, and I have a lot of moles.

P86, female, age 57 years

#### Empowerment

A significant number of participants expressed a desire to enhance their knowledge, understanding, and autonomy regarding their personal health. They frequently perceived participation in the trial as an opportunity to learn about melanoma, which they considered both a motivator (eg, P1, female, age 47 years: *“I would like to be more educated”*) and an expectation (eg, P41, male, 34 years: *“better knowledge of melanoma”*). Participants anticipated improving and gaining confidence in their SSE skills. They believed that by enhancing these skills, they would become more aware of their skin and potential changes, ultimately leading to greater confidence in their ability to perform SSE effectively. As one participant explained:

I hope to learn to recognise the signs of a changing mole or spots…and be more confident about it.

P29, female, age 54 years

Many participants described a desire to take a more active role in managing their own health care. For example, a participant commented,

I’m all for further advancement in the ability to do things better for myself - If I can be more in tune with my body and what's happening to it, good or bad, it can only be a good thing.

P10, female, age 48 years

Meanwhile, P20, female, age 50, wanted “*to do the most I can to manage my own health.*” Some patients hoped to become more motivated to check their own skin and to develop a more disciplined routine, such as a participant who said, *“[This is] a good way for me to make regular home checking a part [of] my routine,”* and P99, male, age 40 years, shared, *“Perhaps it may lead to a more diligent skin check regime by moi?”* [P17 female, age 57 years].

#### Additional Care: Access to Telehealth

Some participants recognized the potential benefits of gaining access to a novel telehealth intervention that would have been otherwise unavailable to them. They appreciated the opportunity for more frequent skin monitoring and the additional access to medical services, such as teledermatologist reviews. A participant (P14, male, age 49 years) shared, “I am hoping it will provide a way of getting suspicious spots checked between my regular visits,” while P37 (male, age 59 years) viewed the intervention as “another tool in the prevention of serious melanoma issues going unchecked.” The potential for closer monitoring was identified by P28, male, aged 65 years, who was drawn to the trial due to the opportunity to have “several sets of eyes on my changing spots & skin issues.” Rural participants identified specific advantages of the intervention for their personal circumstances. For instance, a participant expressed:

Due to the distance I live from the [Hospital] in Sydney, it makes sense to perform a self-diagnosis and be able to update it to the app for a professional doctor to examine and determine if there is a reason for me to travel to the [Hospital] for further investigations.

P42, male, age 46 years

#### Better Health Outcomes

Many participants viewed trial involvement as an opportunity for earlier diagnosis and prompt treatment of recurrent melanoma. A participant (P6, female, age 63 years) explained that the intervention “may assist in identifying any further lesions needing attention in a timely manner.” This sentiment was shared by others who valued the possibility of early detection:

To increase the possibility of finding melanoma early” and “To have more chance of early detection and treatment if I get more melanoma.”

P45, female, age 62 years

Many patients, such as P27 (female, age 35 years), were determined to “do whatever I can to prevent further melanomas or catch them early.” Participants perceived that the intervention may facilitate “a more timely response to new skin cancers,” as noted by P14 (male, age 49 years), and assist patients, like P88 (male, age 74 years), to “get early treatment if I found anything suspicious.” Furthermore, the perceived benefits of the intervention extended beyond early detection and treatment to include improved survival rates and overall quality of life for patients. As a participant expressed, “I hope to catch melanomas before they kill me” [P13, female, age 61 years].

#### Reassurance

For some participants, trial participation offered a sense of reassurance, which was closely related to themes such as receiving additional care and achieving improved health outcomes. They believed that engaging in the trial would give them “peace of mind” as they had taken additional measures to minimize the risk of recurrence. As a participant (P18, female, age 49 years) stated, “[I will feel] more confident that I have not had a recurrence.” This was echoed by P3 (male, age 84 years), who sought “peace of mind regarding …any future melanomas.” In addition, the trial offered participants such as P65 (male, age 50 years) an opportunity to quickly address potential concerns, such as “Ease of mind that if I do identify something, I can send it through for assessment.” Overall, trial participation was seen as a means to instill confidence and reassurance in the pursuit of better health outcomes.

### Theme 3: Trust in Health Practitioner or Facility

Physician influence was identified as a major determinant for trial participation, as illustrated by 21 responses to the first question. Notably, this theme was not present in the expectations voiced in response to the second question. Often, patients enrolled in the trial due to their doctor’s request or recommendation, emphasizing the significance of trust in their treating clinician. For instance, P3 (male, age 84 years) mentioned, “I hold my doctor in high regard, and he recommended it.”

In addition, several respondents with positive experiences conveyed gratitude and a desire to “give back” to the health care facility where they had received treatment.

A participant described trial participation as:

One way for me to give back to the team that have cared for me at [Facility] as they have always demonstrated care, compassion, and empathy.

P32, female, age 62 years

This underscores the impact of personal experiences and trust in medical professionals on patients’ willingness to participate in clinical trials.

## Discussion

### Principal Findings

This SWAT provides valuable insights into patients’ motivations for and expectations from participating in the MEL-SELF RCT of patient-led melanoma surveillance using patient-performed mobile teledermoscopy. Common overarching themes related to benefitting the community by contributing to medical research or helping others, personal benefit through improved health outcomes, and trust in the clinician’s request. These themes were consistently voiced by participants with diverse sociodemographic and clinical characteristics, including those who completed the baseline questionnaire but did not complete the active run-in. While in keeping with identified motivators for trial participation in other conditions [8], this sample of melanoma patients reported distinct, notable influencing factors. Community benefit was the most frequently cited reason for participation (n=67), highlighting a significant yet underappreciated desire among this population to contribute positively to their community. Subthemes specific to the MEL-SELF trial context included a strong emphasis on self-empowerment and a specific interest in the merits of patient-led surveillance, particularly related to telehealth and increasing health care access for people living in rural and remote areas. Understanding motivators in a specific trial context is useful, as factors influencing participation may vary based on patient population, setting, intervention, and disease. The proportions of participants expressing the common overarching themes may also differ according to context. For example, patients with advanced melanoma may be more willing to join clinical trials seeking improved treatment options, while early-stage melanoma patients, as in our trial, were primarily motivated by the desire to help others with melanoma.

Most participants acknowledged the importance of research and expressed motivation to help others, often referencing a sense of social responsibility. In addition, participants demonstrated a clear understanding that medical practice relies on clinical trial outcomes and anticipated direct translation of results into clinical practice. Consequently, many opted to participate with the intention of contributing to the development of new management options for melanoma in the hope this will benefit future patients. Participants identified personal health benefits from taking part in the trial, including increased melanoma knowledge and SSE skills, a more active role in their health care, increased self-efficacy, potentially earlier diagnosis and treatment of subsequent melanomas, and early access to an innovative intervention. It is important to note that most of these perceived personal benefits would only be available to those subsequently randomized into the intervention group. To ensure awareness of equal allocation chances, a final step where participants explicitly acknowledge this was implemented before randomization.

The strengths of our study include conducting the survey prospectively at the point of enrolment, which minimized recall bias and allowed for an accurate assessment of participants’ initial motivations and expectations. In addition, enrolling consecutive patients prevented selection bias, ensuring a representative sample of trial participants. We used rigorous methods in accordance with best practices in qualitative research. There are also several limitations. While our sample is representative of the MEL-SELF population and helps answer our research question within this context, it is primarily composed of highly educated individuals, a majority of whom are women from metropolitan areas with high socioeconomic status. Consequently, our findings may not be generalizable to other populations. In addition, our findings may not apply to other settings, such as higher-risk pharmaceutical trials, but our adaptable methodology could be used in these settings. Furthermore, the extent to which stated motivators and expectations at trial commencement translate into long-term adherence to trial processes remains uncertain. A lengthy trial with a significant participant burden for insufficient gains may lead to diminishing motivation and retention. Finally, our data collection was limited to participants, precluding insights into reasons for nonparticipation. Although our analysis of people who completed the baseline questionnaire but not the active run-in indicated similar findings to the included sample, these may differ for people who did not participate at all. Future research could include interviews with patients who did not participate, particularly those from regional and remote areas and those with indicators of lower socioeconomic status, to identify what would motivate them to engage in research.

### Key Practice Implications

Understanding the factors influencing patient engagement in clinical trials may enable trialists to develop more effective, patient-centered strategies to improve recruitment, response to trial tasks, retention, and adherence to trial interventions. Our SWAT approach, easily implementable in other clinical trials, provides evidence to guide the development of targeted strategies for enhancing trial tasks and processes. The framework of commonly identified motivators could serve as a starting point for other trialists, who may either adopt the existing framework or conduct a SWAT themselves to uncover context-specific themes. Qualitative research is increasingly recognized as a valuable complement to other research methods in dermatology [16]. Although it may seem time-consuming and daunting to some trialists, understanding the underlying factors driving patient participation could ultimately boost trial efficiency and effectiveness.

The findings of this study, together with those of 2 scoping reviews [17,18], have informed refined communication with participants and improved study materials in the MEL-SELF trial, which may, in turn, improve recruitment and consent processes, response rates, retention, and adherence (Figure 1, Table S2 in Multimedia Appendix 1). We implemented strategies targeting clinician influence, such as providing feedback to clinicians on their role in motivating trial participation, sending participant reminders to complete trial tasks from the treating clinician rather than the researcher, and reminding clinicians to discuss trial involvement with participating patients at follow-up visits. In addition, feeding back to recruiting clinicians the diverse reasons reported for trial participation presents an opportunity to enhance recruitment and the consent process by explaining the perceived benefits of participation in alignment with value statements summarized by health agencies [19,20]. Mapping determinants to behavior change theory, such as the Theoretical Domains Framework (TDF) and Capability, Opportunity, Motivation-Behavior (COM-B) model may improve understanding of decision-making and offer guidance for strategies to improve trial processes [21]. We have tailored ongoing MEL-SELF trial communication strategies (participant conversations and reminders) to address patient motivations and will evaluate the effects of these in a future SWAT.

### Conclusion

This SWAT revealed context-specific motivators for trial engagement (a prevalent desire to benefit the community, empowerment, and perceived telehealth benefits), which can be used to tailor communication and study materials in the current MEL-SELF trial and future trials.

Assessing participant motivations through surveys may refine research planning and enhance trial processes in different clinical research settings.

## Appendix

Multimedia Appendix 1 Supplementary tables.

## Abbreviations

COM-B: Capability, Opportunity, Motivation-Behavior model
PRioRiTy: Prioritising recruitment and retention in randomised trials
RCT: randomized clinical trial
REDCap: Research Electronic Data Capture
SSE: skin self-examination
SWAT: Study Within A Trial
TDF: Theoretical Domains Framework

## Data Availability

The data that support the findings of this study are available on request from the corresponding author.

## References

[1] Chalmers I, Glasziou P (2009). Avoidable waste in the production and reporting of research evidence. Lancet.

[2] Treweek S, Bevan S, Bower P, Campbell M, Christie J, Clarke M, Collett C, Cotton S, Devane D, El Feky A, Flemyng E, Galvin S, Gardner H, Gillies K, Jansen J, Littleford R, Parker A, Ramsay C, Restrup L, Sullivan F, Torgerson D, Tremain L, Westmore M, Williamson PR (2018). Trial forge guidance 1: what is a study within a trial (SWAT)?. Trials.

[3] Treweek S, Pitkethly M, Cook J, Fraser C, Mitchell E, Sullivan F, Jackson C, Taskila TK, Gardner H (2018). Strategies to improve recruitment to randomised trials. Cochrane Database Syst Rev.

[4] Gillies K, Kearney A, Keenan C, Treweek S, Hudson J, Brueton V, Conway T, Hunter A, Murphy L, Carr PJ, Rait G, Manson P, Aceves-Martins M (2021). Strategies to improve retention in randomised trials. Cochrane Database Syst Rev.

[5] Houghton C, Dowling M, Meskell P, Hunter A, Gardner H, Conway A, Treweek S, Sutcliffe K, Noyes J, Devane D, Nicholas JR, Biesty LM (2020). Factors that impact on recruitment to randomised trials in health care: a qualitative evidence synthesis. Cochrane Database Syst Rev.

[6] Healy P, Galvin S, Williamson PR, Treweek S, Whiting C, Maeso B, Bray C, Brocklehurst P, Moloney MC, Douiri A, Gamble C, Gardner HR, Mitchell D, Stewart D, Jordan J, O'Donnell M, Clarke M, Pavitt SH, Guegan EW, Blatch-Jones A, Smith V, Reay H, Devane D (2018). Identifying trial recruitment uncertainties using a James Lind Alliance priority setting partnership - the PRioRiTy (Prioritising Recruitment in Randomised Trials) study. Trials.

[7] Brunsdon D, Biesty L, Brocklehurst P, Brueton V, Devane D, Elliott J, Galvin S, Gamble C, Gardner H, Healy P, Hood K, Jordan J, Lanz D, Maeso B, Roberts A, Skene I, Soulsby I, Stewart D, Torgerson D, Treweek S, Whiting C, Wren S, Worrall A, Gillies K (2019). What are the most important unanswered research questions in trial retention? A James Lind Alliance priority setting partnership: the PRioRiTy II (Prioritising Retention in Randomised Trials) study. Trials.

[8] Sheridan R, Martin-Kerry J, Hudson J, Parker A, Bower P, Knapp P (2020). Why do patients take part in research? An overview of systematic reviews of psychosocial barriers and facilitators. Trials.

[9] Ackermann DM, Smit AK, Janda M, van Kemenade CH, Dieng M, Morton RL, Turner RM, Cust AE, Irwig L, Hersch JK, Guitera P, Soyer HP, Mar V, Saw RPM, Low D, Low C, Drabarek D, Espinoza D, Emery J, Murchie P, Thompson JF, Scolyer RA, Azzi A, Lilleyman A, Bell KJL (2021). Can patient-led surveillance detect subsequent new primary or recurrent melanomas and reduce the need for routinely scheduled follow-up? A protocol for the MEL-SELF randomised controlled trial. Trials.

[10] Ackermann DM, Dieng M, Medcalf E, Jenkins MC, van Kemenade CH, Janda M, Turner RM, Cust AE, Morton RL, Irwig L, Guitera P, Soyer HP, Mar V, Hersch JK, Low D, Low C, Saw RPM, Scolyer RA, Drabarek D, Espinoza D, Azzi A, Lilleyman AM, Smit AK, Murchie P, Thompson JF, Bell KJL (2022). Assessing the potential for patient-led surveillance after treatment of localized melanoma (MEL-SELF): a pilot randomized clinical trial. JAMA Dermatol.

[11] Ackermann D, Drabarek D, Hersch J, Bracken K, Turner R, Janda M, Bell K SWAT 190: Identifying motivators of participation and key expectations of participants in randomised trials.

[12] Andresen EL, Wilson KA, Castillo A, Koopman C (2010). Patient motivation for participating in clinical trials for depression: validation of the motivation for clinical trials inventory-depression. Int Clin Psychopharmacol.

[13] Arnetz J, Sudan S, Goetz C, Arnetz B, Gowland L, Manji S, Ghosh S (2019). Preliminary development of a questionnaire measuring patient views of participation in clinical trials. BMC Res Notes.

[14] Weber R (1990). Basic Content Analysis.

[15] Hsieh HF, Shannon SE (2005). Three approaches to qualitative content analysis. Qual Health Res.

[16] Pascual MG, Morris MA, Kohn LL (2023). Publication trends of qualitative research in dermatology: a scoping review. JAMA Dermatol.

[17] Ackermann DM, Bracken K, Janda M, Turner RM, Hersch JK, Drabarek D, Bell KJL (2023). Strategies to improve adherence to skin self-examination and other self-management practices in people at high risk of melanoma: a scoping review of randomized clinical trials. JAMA Dermatol.

[18] Ackermann D, Bracken K, Hersch J, Drabarek D, Janda M, Turner R, Bell K (2021). Strategies to improve participant recruitment, retention, response, and intervention adherence in randomised controlled trials of early detection in populations at high risk of melanoma: a scoping review protocol. OSF HOME.

[19] (2018). Consumer guide to clinical trials.

[20] The value of clinical trials.

[21] Michie S, van Stralen MM, West R (2011). The behaviour change wheel: a new method for characterising and designing behaviour change interventions. Implement Sci.

