# Supplementary material for: Participant Motivators and Expectations in the MEL-SELF Randomized Clinical Trial of Patient-Led Surveillance for Recurrent Melanoma: Content Analysis of Survey Responses
Source: JMIR Dermatol. 2024 Oct 17;7:e58136. doi: 10.2196/58136 (PMC11528161; doi:10.2196/58136)
Supplement: Multimedia Appendix 1 [file derma_v7i1e58136_app1.docx]

**Table S1. Illustrative quotes for identified themes and subthemes for research engagement in the MEL-SELF randomized clinical trial**

| **Category** | **Themes** | **Description and Quotes** |
| --- | --- | --- |
| **Community benefit** | **Contribution to scientific research** | - **Participants broadly recognise the importance of medical research.** - *I am a strong believer in medical research. (P35, female, age 52)* - *I think research is the way forward. (P74, male, age 70* - *Aware of importance of medical research (P83, male, age 70)* - *I have a science background and value research. (P86, female, age 57)* - **Participants link research to improved health outcomes** - *I believe it is important to be a part of research as this will lead to better health outcomes for future patients. (P32, female, age 62)* - *Scientific research and evidence are important for the whole community. It is important to try new techniques to improve treatments. (P56, female, age 64)* - *I believe that medical trials are a major part of development in successful medical treatments. (P58, female, age 60)* - *Research is important to find better ways to approach and treat cancer. (P67, female, age 50)* - **“Sense of duty”**   - *I feel that if I qualify for a medical study, it is my duty to participate. (P78, female, age 73)*   - *As an ex-health services researcher I am interested and feel some responsibility to support research. (P85, male, age 73)* - **May express personal satisfaction arising from their contribution** - *Just the satisfaction from participating (P53, male, age 81)* - *If my input/data can assist this process, I'm grateful to be able to participate. (P58, female, age 60)* |
|  |  | - **Participants specify a wish to contribute to advances in melanoma-related research.** - *To contribute to findings that may inform future prevention and/or management of melanomas (P15, female, age 55)* - *To actively support research into a health issue which has directly impacted me and could again in the future. (P18, female, age 49)* - *to be part of the future of melanoma health care. (P20, female, age 49)* - *Anything to help researchers found out more about melanoma and treatments. (P95, male, age 57)* - ***Feel that their personal experience may particularly benefit others*** - *With my unusual number of melanomas, my experience may provide a clue or two that may lead to greater understanding of skin cancers. (P7 male, age 66)* - *As I am in the high-risk category perhaps my participation would be helpful to others (P70, male, age 80)* |
|  | **Specific merits of the intervention** | - **Telehealth and digital technology research.** - *I support the use of technology as we move forward and potentially into greater endeavours in telehealth. (P98, female, age 69)* - *Technology as a positive and useful tool. (P16, female, age 59)* - *If I can be a part of something that is going drive technology and innovation in this space forward, then I want to be a part of it. (P38, male, age 38)* |
|  |  | - **Benefits people who live in remote areas** - *Seems like a great way to improve the identification of melanoma especially for those who live remotely. (P65, male, age 50)* |
|  |  | - **General** - *I thought the study had value (P59, female, age 58)* - *a way that helped reduce the strain on the health system (P97, male, age 61)* - *Totally believe in the concept. Have great interest in the advances made re Cancer treatment. Very worthwhile in a community spirit sense. (P100, female, age 71)* |
|  | **Altruism** | - **The trial results may benefit future melanoma patients. (i.e. people with a similar experience to themselves)** - *If it helps others who get/have melanoma and their timely diagnosis and treatment is afforded, then I have done something…(P6, female, age 63)* - **May specify future generations of family**   - *Taking part in the study and knowing the results may help others in the future is something I am happy to do. I have a red-headed granddaughter and who knows, she may benefit in the future. (P63, female, age 78)*   - *contribute to research in the hopes more is known for future generations, and my kids. (P27, female, age 35)*   - *There is a strong family history of melanoma and children with fair skin, so this research may help them in the future. (P71, female, age 49)* |
|  |  | - **Broader altruistic comments** - *In the hope that I will help others. (p75, female, age 57)* - **Sometimes expressed together with “nothing for me” in expectation question**   - *Not much really! I just hope it will be of help to others in the future. (P35, female, age 52)*   - *Nothing personally other than looking to assist. (P40, male, age 67)*   - *To help the research…no personal gain (P46, female, age 64)* |
| **Personal benefit** | **Perceived Melanoma Risk** | - **Beneficial due to high-risk status (reason for participation, not expressed in expectations.)** - *I believe I have a real risk of developing melanoma - both my father and older brother had multiple melanomas removed, we spent a lot of time in the sun, unprotected, as children, and I have a lot of moles. (P86, female, age 57)* - *I have had 4 melanomas excised in the last 4 years. (P11, female, age 63)* |
|  | **Empowerment** | - **Increased knowledge, opportunity for learning** - *Better knowledge and understanding of melanoma. (P58, female, age 60)* - *Better knowledge of melanoma (P41, male, age 34)* - *Better understanding of melanoma and what to look out for. (P68, male, age 37)* |
|  |  | - **Improved SSE skills**   - *I hope to gain the skills to check my own skin more thoroughly. (P11, female, age 63)*   - *Improved ability to do skin checks (P15, female, age 55)*   - *I hope the study will give me the tools to check my skin with more confidence (P33, male, age 32)*   - *I want to learn to better spot melanomas. (P41, male, age 34)* - **Improved awareness of skin**   - *Greater awareness of my body (P17, female, age 57)*   - *More awareness of my moles (P24, female, age 42)*   - *I feel that it will heighten my awareness of any potential issues with my skin (P59, female, age 58)* - **Self-confidence in skills**   - *To recognise the signs of a changing mole or spots…and be more confident about it.* *(P29, female, age 54)*   - *To get better at identifying potential issues for myself (P23, female, age 50)*   - *I hope the study will give me the tools to check my skin with more confidence. (P33, male, age 32)*   - *Be better equipped and confident in early self-diagnosis (P57, female, age 57)*   - *More confidence with checking my own skin for early changes. (P71, female, age 49)* |
|  |  | - **Increased self-management: more active role in own health care.** - *I'm all for further advancement in the ability to do things better for myself- If I can be more in tune with my body and what's happening to it good or bad it can only be a good thing. (P10, female, age 48)* - *To do the most I can to manage my own health (P20, female, age 49)* - *get better at identifying potential issues for myself. (P23, male, age 50)* - *any additional help i can get to monitor my own skin is a bonus (P25, male, age 47)* - **Motivation and establishment of routine** - *a good way for me to make regular home checking a part if my routine (P17, female, age 57)* - *Will give me the opportunity to get back into a good skin check routine (P93, female, age 65)* - *I like the idea of a structured way for me to check myself more regularly (P18, female, age 49)* - *Perhaps it may lead to a more diligent skin check regime by moi? (P99, male, age 40)* |
|  | **Additional care: telehealth** | - **Additional access to medical services (teledermatologist review)** - *I am hoping it will provide a way of getting suspicious spot checked between my regular visits. (P14, male, age 49)* - *Quicker diagnoses and answers on suss moles instead of having to wait months till my next skin check. (P9, female, age 37)* - *I … liked the idea of the app to compare my moles rather than trusting mine or my husband's untrained eye (P18, female, age 49)* - *Several sets of eyes on my changing spots & skin issues (P28, male, age 65)* - *I think it is another tool in the prevention of melanoma issues going unchecked (P37, male, age 59)* - *Would be helpful to know if I have a concern that someone may review it without having to book an appointment earlier for a physical exam (P39, female, age 42)* - *it sounded like a good way to stay in touch even more to hopefully monitor my moles even better. (P19, female, age 39)* - *another tool in the prevention of serious melanoma issues going unchecked (P37, male, age 59)* |
|  |  | - **Specific advantages for rural patients** - *due to the distance I live from the RPA in Sydney, it makes sense to perform a self-diagnosis and be able to update it to the app for a professional doctor to examine and determine if there is a reason for me to travel to the RPA for further investigations. (P42, male, age 46)* - *Would be great to be able to choose to be in the intervention group and access the new technology, especially as I live rurally and have such frequent skin checks. (P27, female, age 35)* |
|  | **Better outcomes** | - **Earlier diagnosis and treatment** - *It may assist in identifying any further lesions needing attention in a timely manner. (P6, female, age 63)* - *To increase the possibility of finding melanoma early* - *To have more chance of early detection and treatment if I get more melanoma. (P45, female, age 62)* - *I am wanting to do whatever I can to prevent further melanomas, or catch them early (P27, female, age 35)* - *Providing a more timely response to any new skin cancers (P14, male, age 49)* - *For myself, it may assist in identifying any further lesions needing attention in a timely manner. (P6, male, age 63)* - *Early detection of potential melanomas (P88, male, age 74)* - *Help to get early treatment if I found anything suspicious. (P88, male, age 74)* |
|  |  | - **Survival and quality of life** - *I hope to catch melanomas before they kill me. (P13, female, age 61)* |
|  | **Reassurance** | - **Reassurance due to perceived personal health benefits** - *Peace of mind. To feel I've done all I could to minimise risk of recurrence* - *More confident that I have not had a recurrence. (P18, female, age 49)* - *Peace of mind re my past melanoma and any future melanomas. (P3, male, age 84)* - *Ease of mind that if I do identify something I can send it through for assessment. (P65, male, age 50)* |
| **Doctor or health facility** | **Relationship** | - **Clinician influence** - *Because Dr [x] asked me if I would like to participate in the study. (P12, male, age 77)* - *I was asked by my melanoma specialist if I would be interested in participating in the study. (P6, male, age 63)* |
|  |  | - **Trust in clinician** - *I hold my doctor in high regard and he recommended it. (P3, male, age 84)* |
|  |  | - **Reciprocity** - *To assist the clinic that has supported my skin health over the previous ten years. (P44, male, age 53)* - *one way for me to give back to the team that have cared for me at The Melanoma Institute as they have always demonstrated care, compassion, and empathy, as well as foremost at the front of medical research in this area. (P32, female, age 62)* - *To help doctors and researchers at melanoma unit (P80, female, age 66)* |

**Table S2. Strategies targeting identified motivators of research engagement implemented in the MEL-SELF trial by trial process.**

| **TRIAL PROCESS** | **APPLICATION** | **TARGETED THEME** |
| --- | --- | --- |
| **RECRUITMENT:** |  |  |
| **Eligibility screen** | Conducted by treating doctor at scheduled visit  Feedback to screeners on identified participant motivations to guide conversations. | Clinician influence  All |
| **Information Sheet** | Reinforces the credibility of the people and organizations involved in the research | Community benefit |
| **Informed consent** | Manage expectations by completing randomisation acknowledgement form |  |
| **Active run-in** | Reminders to complete tasks sent from clinician rather than researcher | Clinician influence |
| **INTERVENTION ADHERENCE:** |  |  |
| **PATIENT-LED ARM** |  |  |
| **Scheduled and unscheduled visits** | Clinicians reminded to discuss ongoing trial involvement with participating patients at clinic visits | Clinician influence |
| **SSE, image upload, teledermatology** | Content of reminders targeting potential personal benefit (evaluated in a SWAT^1^). | Personal Health benefit |
| **CLINICIAN-LED ARM** |  |  |
| **Scheduled and unscheduled visits** | Clinicians reminded to discuss ongoing trial involvement with participating patients at clinic visits | Clinician influence |
| **RESPONSE TO TRIAL TASKS:** |  |  |
| **6 monthly questionnaires** | Non responders reminded of their own specific motivations and expectations from trial involvement.  Clinician name included with reminders | Community benefit  Clinician influence |
| **Patient diaries** | SWAT: content of reminders targeting community benefit.  Non responders reminded of their own specific motivations and expectations from trial involvement.  Clinician name included with reminders | Community benefit  Community benefit  Clinician influence |

^1^ SWAT: Study Within A Trial
